# Comparative Analysis of the Oral Frailty Five-item Checklist and Oral Frailty Index-8 Tools in Assessing Oral Frailty and Their Association with Systemic Health Indicators

**DOI:** 10.31662/jmaj.2025-0057

**Published:** 2025-08-22

**Authors:** Hiroshi Kusunoki, Shotaro Tsuji, Kazumi Ekawa, Nozomi Kato, Keita Yamasaki, Fumiki Yoshihara, Hideo Shimizu

**Affiliations:** 1 Department of Internal Medicine, Osaka Dental University, Hirakata, Japan; 2 Department of Hypertension and Nephrology, National Cerebral and Cardiovascular Center, Suita, Japan; 3 Tsuji Surgical Rehabilitation Hospital, Osaka, Japan; 4 Department of Preventive Medicine, Hyogo Medical University Faculty of Medicine, Nishinomiya, Japan; 5 Medical Corporation Shimanami, Nishinomiya, Japan

**Keywords:** oral frailty, frailty, Oral Frailty Five-item Checklist (OF-5), Oral Frailty Index-8 (OFI-8)

## Abstract

**Introduction:**

Oral frailty, defined as an age-related decline in oral function, represents a significant risk factor for adverse health outcomes, though it can be mitigated through early intervention. The Oral Frailty Five-item Checklist (OF-5), introduced in 2023, assesses oral frailty using 5 indicators: reduced number of teeth, difficulty chewing, difficulty swallowing, dry mouth, and low articulatory oral motor skills. Designed for use beyond dental clinics, the OF-5 has demonstrated predictive validity for physical frailty and mortality. Similarly, the Oral Frailty Index-8 (OFI-8) comprises 8 items evaluating oral health, social participation, and dental habits.

**Methods:**

This study compared the OF-5 and OFI-8 tools and investigated their associations with physical and biological markers. A cross-sectional analysis was conducted on 270 Japanese participants aged ≥65 years (median age: 78 years). The assessments included blood tests, physical measurements, and grip strength evaluation. Participants were categorized by sex and oral frailty risk based on OF-5 scores (non-frailty: ≤1; frailty: ≥2).

**Results:**

Oral frailty, defined as an OF-5 score ≥2, was observed in 40.7% of the participants (33.8% in men and 47.4% in women). Both the OF-5 and OFI-8 scores were higher in women than in men. Sex-specific differences in physical and biological markers were evident; men exhibited higher grip strength, whereas women had a higher prevalence of anemia and osteoporosis. Women were also more likely to report reduced masticatory ability and lower levels of social participation. A high concordance rate of over 80% was observed between oral frailty (OF-5 score ≥2) and high-risk oral frailty (OFI-8 score ≥4).

**Conclusions:**

These findings highlight the utility of subjective questionnaires in assessing oral frailty and emphasize the need for longitudinal studies to evaluate their predictive accuracy for physical frailty.

## Introduction

“Oral frailty,” first proposed by the Japanese Geriatrics Society in 2014, is defined as an age-related decline in oral function. It is characterized by an overlap of minor reductions in dental and oral capabilities that may increase the risk of adverse health outcomes ^(1)^. Although oral frailty elevates the risk of further deterioration ^(2)^, it remains reversible with timely and appropriate interventions. Evidence also suggests that physical, social, psychological, and cognitive dimensions of frailty may contribute to the development of oral frailty by reducing individuals’ capacity for self-care and limiting access to adequate professional oral care ^(3), (4), (5)^. Therefore, interventions for oral frailty should be part of a comprehensive approach that recognizes it as one aspect of multidimensional frailty ^(6)^.

In 2023, the Oral Frailty Five-Item Checklist (OF-5) was introduced as a new diagnostic criterion for oral frailty ^(7)^. The OF-5 includes 5 components: fewer teeth, chewing difficulty, swallowing difficulty, dry mouth, and low articulatory oral motor skills. The original OF-5 study evaluated chewing difficulty, swallowing difficulty, and dry mouth using subjective questionnaires, whereas objective assessments were employed for tooth count and articulatory oral motor skills. It is reported that the concordance between subjective and objective evaluations was high ^(8)^. A consensus statement released in 2024 by the Japanese Geriatrics Society, Japanese Geriatric Dentistry Society, and Japanese Society for Sarcopenia and Frailty suggested replacing the objective assessment of articulatory oral motor skills with the self-reported question, “Have you had difficulty with clear pronunciation recently?” It also recommended allowing self-reported tooth counts (>20 teeth) ^(9)^. However, further research is required to determine whether self-reported OF-5 assessments can accurately predict physical frailty outcomes.

Designed for applications beyond dental clinics, the OF-5 is a versatile tool suitable for use by non-dental healthcare professionals and community members. It has demonstrated strong predictive validity for physical frailty, impairment, and mortality among older adults in Japan ^(7)^.

Our recent study involving older adults from the Sasayama-Tamba area of Hyogo Prefecture (Frail Elderly in Sasayama-Tamba Area: FESTA study) demonstrated that oral frailty, diagnosed using the OF-5, was associated with systemic frailty and sarcopenia indicators. Additionally, it was correlated with the progression of systemic frailty during follow-up ^(10)^. However, few studies have reported findings from the application of the OF-5 in urban general internal medicine outpatient settings. Moreover, while our previous study employed objective evaluations of tooth count and oral motor skills, few studies have utilized self-reported assessments as proposed in the 2024 statement.

In contrast, the Oral Frailty Checklist/Oral Frailty Index-8 (OFI-8), developed by the Japan Dental Association, comprises 8 items: (1) chewing difficulty, (2) swallowing difficulty, (3) denture use, (4) dry mouth, (5) reduced frequency of outings, (6) difficulty chewing hard food, (7) tooth brushing at least twice daily, and (8) regular dental visits. Items (1)-(3) are scored as 2 points each, whereas the remaining items are scored as 1 point each, with a maximum score of 11. Scores are categorized as low risk (0-2 points), moderate risk (3 points), or high risk (>4 points). The Cox proportional hazards model revealed that participants with a score of 3 points had a 2.1-fold risk of new-onset oral frailty. Similarly, those with a score of 4 or more had a 3.1-fold risk of new-onset oral frailty and a 1.4-fold risk of needing long-term care. When treated as a continuous variable, a 1-point increase in the OFI-8 score corresponded to a 1.3-fold increase in the risk of new-onset oral frailty and a 1.1-fold increase in the risk of requiring long-term care ^(11)^.

In our previous study on the OFI-8, the OFI-8 questionnaire was administered to patients attending a general internal medicine outpatient clinic ^(12)^. The OFI-8 scores were higher in women than in men, suggesting that oral frailty is more prevalent among women. In the high-risk group for oral frailty (OFI-8 score ≥4), grip strength was significantly lower in both men and women. Additionally, among men, height, hemoglobin levels, red blood cell count, and serum albumin levels were significantly lower in those with an OFI-8 score ≥4.

Both OF-5 and OFI-8 are based on the Kashiwa Study, a longitudinal prospective cohort study conducted in the Tokyo metropolitan area ^(7), (11)^. In addition, OF-5 has been used in the Otassha Study, which targeted community-dwelling residents in Itabashi Ward, Tokyo ^(8)^. As for outpatient populations, OF-5 has also been applied to patients attending the Frailty Outpatient Clinic at the National Center for Geriatrics and Gerontology Hospital in Ōbu, Aichi Prefecture ^(13)^.

It should be noted that community-dwelling older adults who participate in epidemiological surveys are subject to selection bias, as they tend to have greater health awareness. Similarly, older adults attending frailty outpatient clinics may have different backgrounds from the general older population. In the present study, we targeted patients attending the general internal medicine outpatient departments of 2 hospitals―a university dental hospital and a cardiovascular specialty hospital―primarily for the management of lifestyle-related diseases. We consider that this study population represents typical older adults living in urban areas of Japan, and thus our findings can be generalized.

Originally, the Kashiwa Study demonstrated the following associations between higher OF-5 scores, physical disability, and mortality: higher scores were associated with an increased risk of physical disability (adjusted hazard ratio: 1.40; 95% confidence interval [CI]: 1.14-1.72) and mortality (adjusted hazard ratio: 1.44; 95% CI: 1.11-1.87) ^(7)^. Furthermore, our FESTA study also indicated that OF-5 is associated with the worsening of physical frailty, as assessed by the J-CHS criteria ^(10)^.

Although the OF-5 and OFI-8 share common items, such as chewing difficulty, swallowing difficulty, and dry mouth, the OF-5 uniquely incorporates objective evaluations of tooth count and oral motor skills. However, the comparative efficacy of the OF-5 and OFI-8 in predicting physical frailty remains underexplored.

The objectives of this study were as follows: 1) To quantify the number of individuals meeting the criteria for each item in the OF-5 and OFI-8, stratified by sex. 2) To calculate the prevalence of oral frailty or high oral frailty risk as determined by each method. 3) To evaluate the concordance rate between the 2 assessment methods. 4) To explore the characteristics of each assessment tool.

Additionally, this study aimed to assess sex differences in physical and blood markers among individuals classified as having oral frailty using the OF-5. Using the OF-5, the study also investigated whether individuals with suspected oral frailty (OF-5 score ≥2) exhibited differences in physical and biological markers, including height, weight, blood indices, and grip strength, compared with those with lower OF-5 scores.

## Materials and Methods

This was a cross-sectional study. All patients aged ≥65 years who were enrolled in this study were Japanese and provided informed consent. A total of 270 patients were enrolled, with a median age of 78 (72-83) years. The cohort included 133 men (median age, 77 years) and 137 women (median age, 79 years) who were admitted to the Osaka Dental University and the National Cerebral and Cardiovascular Center between February 2024 and September 2024.

The participants in this study were outpatients, not inpatients. They regularly visited the clinic for the management of lifestyle-related diseases such as hypertension and diabetes. To minimize potential confounding factors, we excluded the following individuals from the study: (1) those who were non-ambulatory or bedridden; (2) those with advanced malignant diseases and a life expectancy of less than one year; (3) those who were unable to complete questionnaire-based interviews due to cognitive impairments such as dementia; and (4) those undergoing maintenance dialysis.

Blood tests, physical measurements (height and weight), grip strength measurements, and OF-5 and OFI-8 questionnaires were administered to all participants. Five internal medicine outpatient physicians performed oral assessments using the questionnaire. The results of the questionnaire were tabulated. The maximum grip strength was measured using a grip strength tester (GRIP‐A; Takei Ltd., Niigata, Japan) ^(12)^.

The study protocol was approved by the Ethics Committees of Osaka Dental University and the National Cerebral and Cardiovascular Center. All procedures involving human participants were performed in accordance with the ethical standards of the institutional and/or national research committee where the studies were conducted (Institutional Review Board approval number 111351 at Osaka Dental University) and with the 1964 Declaration of Helsinki and its later amendments or comparable ethical standards. The requirement for written informed consent was waived, as participants were given the opportunity to opt out due to the retrospective nature of the study.

### Questionnaires

The OF-5 and OFI-8 were used in this study. The following 10 questions were asked to all participants, including 3 common questions shared by the OF-5 and OFI-8 (Table 1). For the OF-5 evaluation, the OF-5 score was determined by the number of applicable items from questions ① through ⑤. Based on the questionnaire results, participants were classified by sex into 2 groups: the oral non-frailty group (scores ≤1) and the oral frailty group (scores ≥2). These classifications were then used for further analysis.

**Table 1.** Questionnaire Items Used in This Study.

OF-5 specific questions	① Fewer teeth
	How many of your natural teeth are left? (<20 natural teeth)
	② Low articulatory oral motor skill
	Have you had difficulty with clear pronunciation recently? (Yes)
Questions common to OF-5 and OFI-8	③ *Difficulty in chewing*
	Do you have any difficulties eating tough foods compared with 6 months ago? (Yes)
	④ *Difficulty in swallowing*
	Have you choked on your tea or soup recently? (Yes)
	⑤ *Dry mouth*
	Do you often experience having a dry mouth? (Yes)
OFI-8 specific questions	⑥ Do you use dentures? (Yes)
	⑦ Do you go out less frequently than you did last year? (Yes)
	⑧ Can you eat hard foods like squid jerky or pickled radish? (No)
	⑨ How many times do you brush your teeth in a day? (<2 times/day)
	⑩ Do you visit a dental clinic at least annually? (No)

OF-5: Oral Frailty Five-item Checklist; OFI-8: Oral Frailty Index-8.

For the OFI-8 evaluation, the standard scoring protocol was applied. Participants received 2 points for each “yes” response to items ③, ④, or ⑥. For items ⑤, ⑦, and ⑨, 1 point was given for each “yes” response. For items ⑧ or ⑩, 1 point was given for each “no” response. The maximum possible score was 11. The screening criterion was defined as the sum of the OFI-8 scores. The higher the OFI-8 score, the higher the risk of oral frailty; that is, 0-2 points indicated a low risk, 3 points indicated a moderate risk, and >4 points indicated a high risk. In this study, participants were categorized by sex, OFI-8 score ≤3 (low-to-moderate-risk group), and OFI-8 score ≥4 (high-risk group). The characteristics of each group were examined. Additionally, the number of applicable items for each question on the OFI-8 and OF-5 was analyzed. Comparisons of blood parameters, physical measurements, and grip strength were conducted between individuals with an OF-5 score of ≤1 and those with a score of ≥2.

### Statistical analysis

The distribution of data was assessed for normality using histograms and the Shapiro-Wilk test. For intergroup comparisons, the Mann-Whitney U test (also known as the Wilcoxon rank-sum test) was used when the assumption of normality was violated, instead of Student’s t-test. Categorical variables are presented as frequencies and percentages, while continuous variables are reported as means ± standard deviations or as medians with interquartile ranges (IQR; first and third quartiles). Inter-rater agreement was evaluated using both observed agreement and Cohen’s Kappa coefficient. Receiver operating characteristic (ROC) analysis was performed to assess the diagnostic performance of the OFI-8 score in predicting OF-5 score ≥2, and the area under the curve (AUC) was calculated. Categorical variables were analyzed using Fisher’s exact test. All statistical analyses were conducted using JMP software version 17.1 (SAS Institute Inc., Cary, NC, USA). p<0.05 was considered statistically significant.

## Results

### Participant characteristics and findings

Male participants were significantly taller, heavier, and exhibited greater grip strength than female participants (all p < 0.001). Hemoglobin levels and hematocrit values were higher in men (p < 0.001), while women exhibited lower hemoglobin and red blood cell counts. Creatinine levels were higher in men than in women, likely reflecting a greater muscle mass. Malignancies were more common in men (p = 0.005), whereas osteoporosis was more prevalent in women (p = 0.035) (Table 2).

**Table 2.** Baseline Characteristics of the Participants Stratified by Sex and the OF-5 and OFI-8 Questionnaires.

		Total (n = 270)	Men (n = 133)	Women (n = 137)	P value
Age (years)‡		78 (72-83)	77 (71-83)	79 (73-83)	0.127
Height (cm)‡		157 (151-165)	165 (160-170)	152 (148-156)	<0.001
Weight (kg)‡		59 (50-68)	65 (60-73)	52 (48-59)	<0.001
Body mass index: BMI‡		23.3 (21.4-25.8)	24.3 (22.3-26.4)	22.5 (20.6-25.3)	<0.001
Grip strength (kg)‡		21.5 (16-29.5)	29.5 (24.7-34.0)	16.6 ± 4.4	<0.001
White blood cells (/μL)‡		5625 (4835-6588)	5850 (4985-6665)	5410 (4795-6545)	0.174
Red blood cells (×104/μL)*		426 ± 52	435 ± 57	417 ± 45	0.004
Hemoglobin (g/dL)‡		13.2 (12.2-14.3)	13.8 (12.8-14.9)	12.8 (11.9-13.6)	<0.001
Hematocrit (%)‡		39.9 (37.2-42.8)	41.6 (38.1-44.6)	38.6 (36.6-41.4)	<0.001
Platelets(×104/μL)*		21.3 ± 5.1	20.5 ± 4.8	22.1 ± 5.3	0.007
Total protein (g/dL)‡		7.0 (6.8-7.3)	7.0 (6.7-7.3)	7.1 (6.8-7.3)	0.098
Albumin (g/dL)‡		4.2 (3.9-4.4)	4.2 (3.9-4.4)	4.2 (3.9-4.4)	0.891
Creatinine (mg/dL)‡		0.85 (0.73-1.05)	0.97 (0.83-1.17)	0.76 (0.66-0.90)	<0.001
Comorbidities					
Hypertension, n (%)†		232 (85.9)	118 (88.7)	114 (83.2)	0.223
Diabetes, n (%)†		60 (22.2)	34 (25.6)	26 (19.0)	0.241
Dyslipidemia, n (%)†		148 (54.8)	66 (49.6)	82 (59.9)	0.112
Osteoporosis, n (%)†		20 (7.4)	5 (3.8)	15 (10.9)	0.035
Malignant neoplasm, n (%)†		23 (8.5)	16 (12.0)	7 (5.1)	0.005
Cardiovascular disease, n (%)†		71 (26.3)	41 (30.8)	30 (21.9)	0.100
Cerebrovascular disease, n (%)†		30 (11.1)	14 (10.5)	16 (11.7)	0.847
Questions					
OF-5 specific questions	① Fewer teeth	90 (33.3)	35 (26.3)	55 (40.1)	0.020
	How many of your natural teeth are left? (<20 natural teeth) †				
	② Low articulatory oral motor skill	31 (11.5)	20 (15.0)	11 (8.0)	0.086
	Have you had difficulty with clear pronunciation recently? (Yes) †				
Questions common to OF-5 and OFI-8	③ *Difficulty in chewing*	56 (20.7)	19 (14.3)	37 (27.0)	0.011
	Do you have any difficulties eating tough foods compared with 6 months ago? (Yes) †
	④ *Difficulty in swallowing*	75 (27.8)	34 (25.6)	41 (29.9)	0.497
	Have you choked on your tea or soup recently? (Yes) †
	⑤ *Dry mouth*	101 (37.4)	46 (34.6)	55 (40.1)	0.380
	Do you often experience having a dry mouth? (Yes) †
OFI-8 specific questions	⑥ Do you use dentures? (Yes) †	156 (57.8)	75 (56.4)	81 (59.1)	0.712
	⑦ Do you go out less frequently than you did last year? (Yes) †	76 (28.1)	28 (21.1)	48 (35.0)	0.015
	⑧ Can you eat hard foods like squid jerky or pickled radish? (No) †	38 (14.1)	13 (9.7)	25 (18.2)	0.054
	⑨ How many times do you brush your teeth in a day? (<2 times/day) †	57 (21.1)	32 (24.1)	25 (18.2)	0.297
	⑩Do you visit a dental clinic at least annually? (No) †	55 (20.4)	25 (18.8)	30 (21.9)	0.549
Questionnaire score results					
OF-5 score‡		1 (0-2)	1 (0-2)	1 (1-2)	0.018
Oral frailty, ≥2 OF-5 score, n (%)†		110 (40.7)	45 (33.8)	65 (47.4)	0.016
OFI-8 score‡		3 (2-5)	3 (2-4)	4 (2-5)	0.016
OFI-8 score ≤2, n (%)†		107 (39.6)	61 (45.9)	46 (33.6)	0.047
OFI-8 score = 3, n (%) †		42 (15.6)	21 (15.8)	21 (15.3)	1.000
OFI-8 score ≥4, n (%)†		121 (44.8)	51 (38.3)	70 (51.1)	0.038

OF-5: Oral Frailty Five-item Checklist; OFI-8: Oral Frailty Index-8. † Presented as n (%). ‡ Presented as the median (25th-75th percentile). * Presented as the mean ± standard deviation. §p-Values for comparisons of study population characteristics between groups with and without oral frailty were calculated using Student’s t test, the Mann-Whitney U test, and categorical variables were analyzed using Fisher’s exact test.

### Oral frailty and functional differences

The overall prevalence of oral frailty, defined as an OF-5 score of ≥2, was 40.7%. The prevalence was significantly higher in women than in men (47.4% vs. 33.8%, p = 0.016). Women were also more likely to report reduced masticatory ability, self-reported difficulty in eating hard foods (p = 0.054), and a decreased frequency of going out (p = 0.015). Additionally, the proportion of individuals with <20 natural teeth was significantly higher among women (p = 0.020) (Table 2).

### OF-5 and OFI-8 scoring analysis

**Figure 1.**
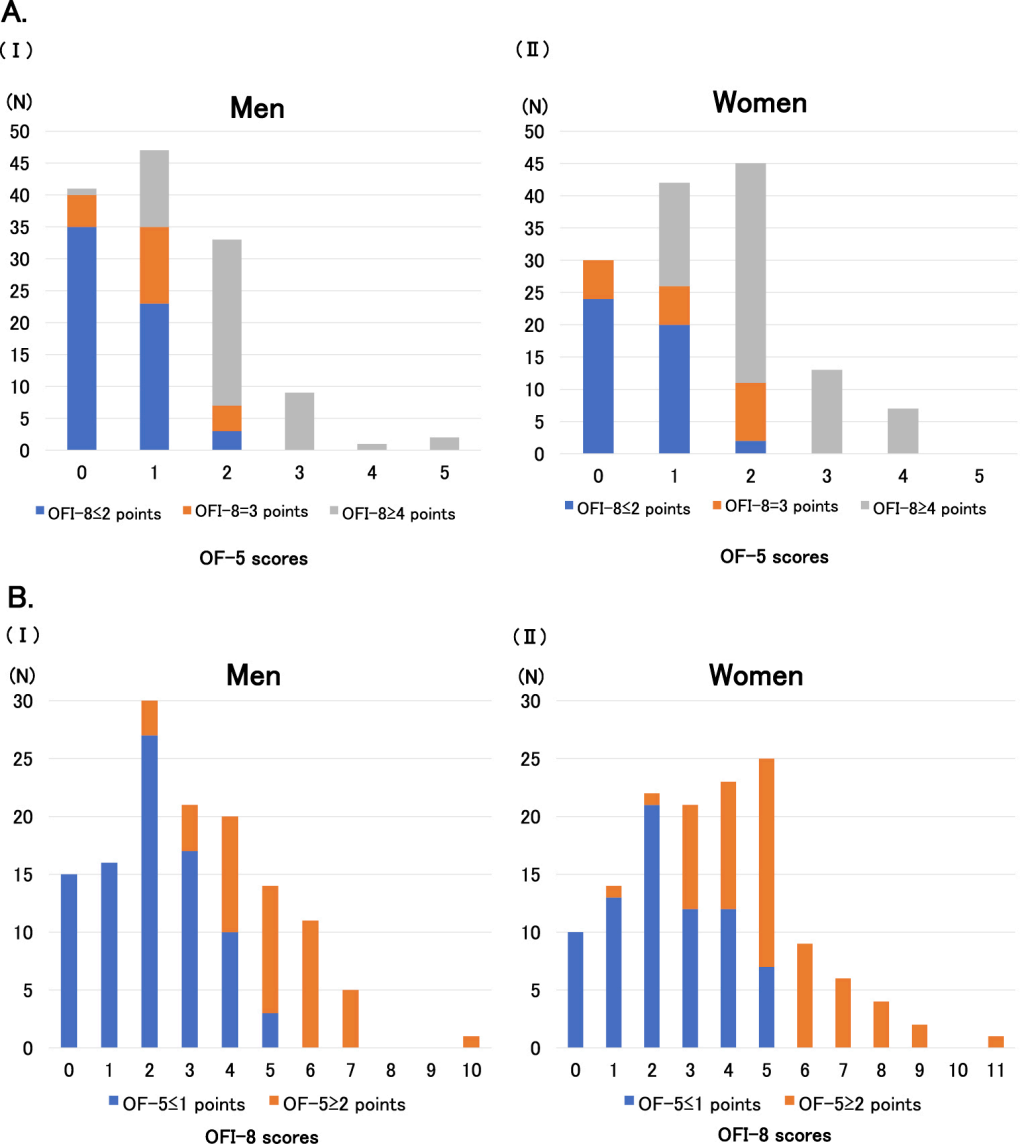
A. Distribution of OF-5 score and their association with OFI-8 in men (Ⅰ) and women (Ⅱ). B. Distribution of OFI-8 score and their association with OF-5 in men (Ⅰ) and women (Ⅱ). OF-5: Oral Frailty Five-item Checklist; OFI-8: Oral Frailty Index-8.

The distribution of OF-5 scores differed by sex, with men most frequently scoring 1 and women most frequently scoring 2. Scores of ≥3 were rare in both sexes. These results suggest that oral frailty is more prevalent in women. For the OFI-8, male scores were concentrated between 2 and 4 points, whereas female scores showed a wider range. The distribution of OFI-8 approached normality, in contrast to that of OF-5. The maximum score for the OFI-8 was 11, whereas the maximum score for the OF-5 was 5 (Figure 1A and B).

**Figure 2.**
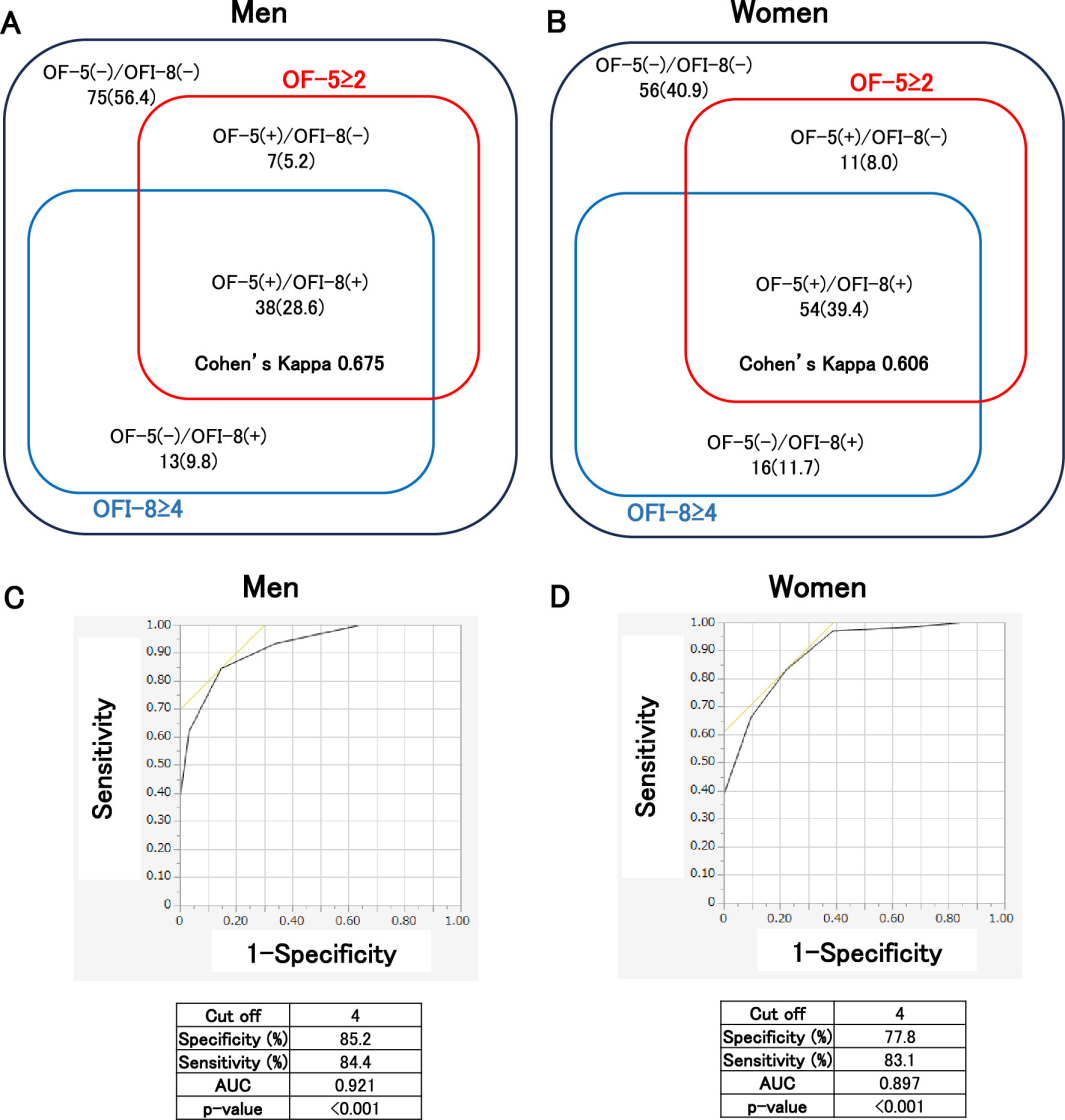
The proportions of individuals with an OF-5 score ≥2 (defined as OF-5(+)) and an OFI-8 score ≥4 (defined as OFI-8(+)) within the overall sample in men (A) and women (B). Notes: x (y), number of patients (%). ROC curves and AUC for OFI-8 score and OF-5 scores ≥2 in men (C) and women (D). AUC: area under the curve; OF-5: Oral Frailty Five-item Checklist; OFI-8: Oral Frailty Index-8; ROC: receiver operating characteristic curve.

The proportions of individuals with an OF-5 score ≥2 (defined as OF-5(+)) and an OFI-8 score ≥4 (defined as OFI-8(+)) within the overall sample, separated by sex, are shown in Figure 2 using a Venn diagram. Among the 45 men diagnosed as OF-5(+), 38 were also diagnosed as OFI-8(+), resulting in 84.4% of OF-5(+) men being OFI-8(+). Among the 65 women diagnosed as OF-5(+), 54 were also diagnosed as OFI-8(+), resulting in 83.1% of OF-5(+) women being OFI-8(+) (Figure 2A and B).

Cohen’s Kappa coefficients representing the agreement between OF-5(+) and OFI-8(+) were 0.675 in men and 0.606 in women (Figure 2A and B). Furthermore, to examine whether the OFI-8 score could predict OF-5(+), a ROC analysis was conducted. High AUC values were observed in both sexes, with an optimal cut-off value of 4 points for both men and women (Figure 2C and D). This cut-off value corresponds to the existing threshold for OFI-8(+): high risk of oral frailty.

### Clinical associations with oral frailty

In men, oral frailty (OF-5 ≥2) was significantly associated with age (p = 0.022) and, unexpectedly, elevated white blood cell counts (p = 0.030). No significant differences were observed in height, weight, grip strength, or other clinical indicators. In women, oral frailty was not significantly associated with age (p = 0.620) (Table 3).

**Table 3.** Comparison of Physical and Biological Markers between the Oral Non-Frailty and Oral Frailty Groups Determined by the OF-5, Stratified by Sex.

	Men (n = 133)			Women (n = 137)		
	Oral non-frailty, 0-1 OF-5 score (n = 88)	Oral frailty, ≥2 OF-5 score (n = 45)	P Value	Oral non-frailty, 0-1 OF-5 score (n = 72)	Oral frailty, ≥2 OF-5 score (n = 65)	P Value
Age (year-old)‡	76 (69-81)	79 (73-85)	0.022	78 (73-83)	79 (74-83)	0.620
Height (cm)*	165.4 ± 7.2	163.5 ± 5.9	0.131	151.9 ± 5.0	151.5 ± 6.8	0.701
Body weight (kg)‡	68 (60-73)	62 (58-71)	0.080	52 (48-60)	52( 45.6-58)	0.410
Body mass index‡	24.6 (22.4-26.6)	23.2 (21.9-26.1)	0.255	22.7 (20.7-25.3)	22.4 (20.5-25.3)	0.449
Grip strength (kg)*	30.2 ± 7.0	28.1 ± 7.3	0.114	17.0 ± 4.1	16.1 ± 4.8	0.248
White blood cells (/μL)‡	5610 (4803-6485)	6300 (5180-7705)	0.030	5370 (4645-6508)	5510 (4840-6560)	0.446
Red blood cells (×10^4^/μL)*	438 ± 59	429 ± 54	0.414	419 ± 42	415 ± 49	0.590
Hemoglobin (g/dL)	13.8 ± 1.7*	13.5 ± 1.4*	0.404	13.0 (11.9-13.7)‡	12.6 (11.9-13.4)‡	0.230
Hematocrit (%)	41.3 ± 4.9*	40.5 ± 4.0*	0.327	39.3 (36.9-41.3)‡	38.2 (36.1-41.5)‡	0.359
Platelets (×10^4^/μL)	20.5 ± 5.0*	20.5 ± 4.5*	0.981	21.3 (18.7-25.2)‡	21.6 (17.9-25.7)‡	0.841
Total protein (g/dL)*	7.0 ± 0.4	7.0 ± 0.5	0.931	7.1 ± 0.4	7.1 ± 0.4	0.957
Albumin (g/dL)‡	4.2 (4.0-4.4)	4.1 (3.9-4.4)	0.241	4.2 (3.9-4.4)	4.1 (3.9-4.3)	0.514
Creatinine (mg/dL ‡	0.95 (0.83-1.19)	0.99 (0.85-1.14)	0.801	0.76 (0.68-0.97)	0.76 (0.64-0.86)	0.381
Hypertension, n (%)†	79 (89.8)	39 (86.7)	0.576	64 (88.9)	50 (76.9)	0.070
Diabetes, n (%)†	22 (25.0)	12 (26.7)	0.836	11 (15.3)	15 (23.1)	0.280
Dyslipidemia, n (%)†	45 (51.1)	21 (46.7)	0.715	47 (65.3)	35 (53.8)	0.222
Osteoporosis, n (%)†	5 (5.7)	0 (0.0)	0.167	5 (6.9)	10 (15.4)	0.170
Malignant neoplasm, n (%)†	10 (11.4)	6 (13.3)	0.782	3 (4.2)	4 (6.2)	0.708
Cardiovascular disease, n (%)†	28 (31.8)	13 (28.9)	0.843	17 (23.6)	13 (20.0)	0.682
Cerebrovascular disease, n (%)†	8 (9.1)	6 (13.3)	0.552	9 (12.5)	7 (10.8)	0.796

OF-5: Oral Frailty Five-item Checklist. † Presented as n (%). ‡ Presented as the median (25th-75th percentile). * Presented as the mean ± standard deviation. §P values for comparisons of study population characteristics between groups with and without oral frailty were calculated using Student’s t test, the Mann-Whitney U test, and categorical variables were analyzed using Fisher’s exact test.

## Discussion

In this study, the oral frailty assessment tools OF-5 and OF-8 were simultaneously administered to patients attending general internal medicine outpatient clinics for lifestyle-related diseases and other conditions. The number of respondents who met the criteria for each question was analyzed, and potential differences in physical indicators were examined. This study demonstrated that these questionnaire-based tools can be effectively and efficiently implemented in general internal medicine settings.

Approximately 40% of patients attending general internal medicine outpatient clinics had an OF-5 score ≥2. The prevalence of oral frailty was similar between men and women, affecting over 40% of the population. The observed 40% prevalence of oral frailty was consistent with several previous studies ^(7), (13), (14)^. Although men generally exhibit better physical function, the progression of oral frailty in men could adversely affect their dietary quality and nutritional intake. Targeted dental care and nutritional guidance for individuals with high OF-5 and OFI-8 scores may help mitigate systemic health risks.

Women were more likely to exhibit reduced masticatory ability and fewer teeth than men, resulting in higher OF-5 scores. Women also reported a lower frequency of going out, potentially indicating the risk of declining mental health and reduced social interactions. Among women, the habit of wearing masks since the COVID-19 pandemic may have increased their reluctance to show their mouth, which could lead them to go out less than before.

Women had higher rates of tooth loss and oral frailty scores, which may be influenced by age-related changes and postmenopausal effects such as osteoporosis. Although the direct association between maintaining bone density and oral function may be debated, osteoporosis is more prevalent in women and is closely linked to systemic frailty and physical decline. Therefore, the observed higher prevalence of oral frailty in women in this study suggests a potential connection between these conditions. Maintaining bone density may help improve oral frailty by supporting overall physical function. Consequently, interventions aimed at preserving bone density and enhancing masticatory function are particularly important in women. Additionally, in patients diagnosed with osteoporosis, bisphosphonates are often prescribed. However, these medications carry a risk of osteonecrosis of the jaw. From the perspective of oral function, careful consideration is needed before initiating pharmacological treatment.

This study also found that women were more likely to experience a decline in chewing ability. Improving this function is therefore desirable. Question ③ (“Do you have any difficulties eating tough foods now compared with 6 months ago?”) and question ⑧ (“Can you eat hard foods like squid jerky or pickled radish?”) both assess chewing ability. A significant sex difference was found for question ③ (p = 0.011), while question ⑧ showed a trend toward significance but did not reach statistical significance (p = 0.054). This may be due to limited statistical power, and a larger sample size might reveal a significant difference for question ⑧ as well.

There are also conceptual differences between the 2 questions: question ③ assesses subjective changes in chewing ability over time, while question ⑧ evaluates current functional ability using specific food items. Subjective assessments like question ③ may be more influenced by sex differences. For example, women may be more sensitive to declines in oral function or more likely to perceive such changes. In contrast, question ⑧, which involves concrete food items, may reflect actual chewing ability more directly, resulting in smaller sex-related differences.

Figure 2 shows the proportions of individuals with an OF-5 score ≥2 (defined as OF-5(+)) and an OFI-8 score ≥4 (defined as OFI-8(+)) among the overall participants. Based on these results, a cut-off of ≥2 points on the OF-5 aligned well with a high-risk classification (≥4 points) on the OFI-8, with over 80% concordance. Cohen’s Kappa coefficients representing the agreement between OF-5(+) and OFI-8(+) were 0.675 in men and 0.606 in women. Based on the ROC analysis, the optimal cut-off value of the OFI-8 score for predicting OF-5(+) was determined to be 4 points in both men and women. This cut-off value corresponds to the existing threshold for OFI-8(+): high risk of oral frailty. This supports the OF-5 cut-off as a valid marker for diagnosing oral frailty and highlights the utility of an OFI-8 score of ≥4 for identifying high-risk individuals.

Over 80% of individuals with an OF-5 score ≥2 were also categorized as high risk for oral frailty with an OFI-8 score ≥4. The OF-5 questionnaire demonstrated ease of use and strong alignment with the OFI-8 for the identification of oral frailty. The OF-5 allows for a simpler evaluation of oral frailty, as it diagnoses oral frailty when 2 of its 5 items are met. In contrast, the OFI-8 includes a mix of 2- and 1-point questions, resulting in uneven weighting among the items, which may occasionally complicate the scoring process. Given the high concordance rate between the OF-5 and OFI-8, the simpler OF-5 tool may be more suitable for use in busy clinical settings. When time is limited, the OF-5, which can screen for oral frailty using only 2 of its 5 items, remains sufficiently useful for screening purposes.

In previous studies, the number of teeth and articulatory oral motor function were objectively assessed by dentists ^(10)^. In the 2024 consensus statement by 3 academic societies, self-reported evaluation was also considered acceptable for assessing these components in the OF-5.

In our previous epidemiological study, the prevalence of low articulatory oral motor skills, as assessed by dentists using oral diadochokinesis (ODK), was relatively high at 36.0% (43.1% in men and 32.4% in women) ^(10)^. However, in the present study, where low articulatory oral motor skills were evaluated based on subjective responses to a questionnaire, the prevalence was significantly lower at 11.5% overall (15.0% in men and 8.0% in women) compared with the ODK-based assessments. For the other 4 items of the OF-5, no substantial differences in prevalence rates were observed between participants in the previous epidemiological study and outpatients in the current study. However, regarding low articulatory oral motor skills, subjective evaluation using a questionnaire appears to underestimate articulatory oral motor skills compared with objective assessment using the ODK. This may be better explained by response bias rather than by an overestimation of ODK.

In contrast, regarding the question of whether the number of teeth is ≥20, previous epidemiological studies relied on measurements conducted by dentists, whereas this study used self-reported data. However, there were no significant differences in the proportion of respondents who met this criterion. These findings suggest that the self-reported assessments of number of teeth are generally accurate.

Our previous study, in which the number of teeth was evaluated by dentists ^(10)^, was conducted on participants from the FESTA study, and its control group differed from that of the present study. The present study focuses on patients attending general internal medicine outpatient clinics in urban hospitals, who are slightly older on average and likely have more comorbidities than those in the FESTA study. However, the proportion of individuals with <20 teeth did not differ significantly between the 2 groups. There was little difference in the number of teeth, regardless of whether it was self-reported or assessed by a dentist.

In our previous study, a longitudinal analysis revealed that initial OF-5 scores predicted increased physical frailty after 2-3 years, particularly in those with higher baseline scores ^(10)^. The OF-5 was a significant factor for frailty progression in both sexes. A longitudinal study is needed to determine whether the assessment of OF-5 using only a questionnaire can serve as a predictor of worsening physical frailty in the future.

Compared to the OF-5, the OFI-8 includes more items, such as those related to social participation and oral hygiene behaviors, which are closely linked to systemic health and daily activities. This broader scope of the OFI-8 may explain its clearer association with physical and blood parameters compared with the OF-5. The OFI-8 has already been shown in a longitudinal study to be significantly associated with the risk of requiring long-term care ^(11)^.

Before the OF-5 was developed, the OFI-8 was published in 2021 and has since been more widely used in practice. The OFI-8 includes items that are not directly related to oral health, such as social participation, and contains more items, allowing for the assessment of a broader range of conditions. In clinical settings, when time permits, it may be beneficial to evaluate all 10 items included in both scales, as this study did. Future research with larger populations could provide further insight into the utility of evaluating all 10 items. Oral frailty assessed using the OFI-8 has been shown to be significantly associated with systemic conditions, such as sarcopenia ^(15)^. More recently, the OFI-8 score has also been reported to correlate with the severity of constipation ^(16)^.

In our previous study using the OFI-8, individuals with an OFI-8 score ≥4, classified as high risk for oral frailty, exhibited significantly lower grip strength and cystatin C-related indices in both men and women. Additionally, in men, albumin and hemoglobin levels were significantly lower when the OFI-8 score was ≥4. Significant associations have been observed between OFI-8 scores and physical as well as blood parameters ^(12)^. In the present analysis, individuals classified as having oral frailty with an OF-5 score ≥2 did not show significantly lower values for grip strength, albumin, or hemoglobin in either men or women.

Epidemiological studies from the FESTA study have shown that albumin and hemoglobin levels are positively associated with muscle mass ^(17), (18)^. Furthermore, lifestyle-related diseases such as hypertension and dyslipidemia are known to be associated with physical frailty ^(19), (20), (21)^. However, no significant differences in these factors were found between participants with and without oral frailty (Table 3). Further research with a larger sample size is needed to determine whether differences in these indicators can be detected.

This study has several limitations that must be acknowledged. First, as this was a cross-sectional study, a cause-and-effect relationship could not be established. A prospective follow-up study is required to assess causal associations between the OF-5 and other indices. Second, this study did not objectively assess oral function using the 7 items specified by the Japanese Society of Gerodontology ^(22)^, leaving the precise oral function of individuals with an OF-5 score ≥2 or an OFI-8 score ≥4 unknown. However, the primary aim of this study was to investigate the prevalence of oral frailty diagnosed using the OF-5 and OFI-8 among patients attending a general internal medicine outpatient clinic and to determine whether oral function assessed via a questionnaire is associated with objective clinical indicators, without relying on specialized dental skills or equipment. Therefore, we believe that the use of professional dental instruments and techniques was not essential for achieving the objectives of this study.

Chinese versions of the OF-5 and OFI-8 have recently been developed and published ^(15), (23)^, reflecting a growing effort to promote the dissemination of these questionnaires beyond Japan. However, as these tools are relatively new, the number of published studies evaluating their application remains limited. A key strength of these questionnaires lies in their simplicity and the feasibility of assessing oral function outside dental clinics, including by patients themselves. Therefore, it is essential to encourage their use in general medical settings and to accumulate further evidence to support their broader implementation.

Older adults with oral frailty have been reported to incur higher medical and dental expenditures, suggesting that oral frailty is not merely a localized oral health issue but may also be associated with the onset and progression of various systemic conditions. Consequently, it is increasingly recognized as a health concern that extends beyond the scope of dentistry ^(24)^. The prevention and early detection of oral frailty may contribute to reducing not only dental care costs but also overall healthcare expenditures, highlighting its importance from the broader perspective of systemic health maintenance ^(25)^.

### Conclusions

The progression of oral frailty varies across sex and age groups, highlighting the importance of providing tailored health support. Future studies should explore the relationship between oral function and systemic health risks to develop effective preventive strategies. The strong concordance between the OF-5 and OFI-8 underscores their complementary utility in identifying individuals at risk for oral frailty. These findings emphasize the need for sex-specific approaches to assess and manage oral health risks in clinical practice.

## Article Information

### Conflicts of Interest

None

### Acknowledgement

We would like to thank all the medical staff of Osaka Dental University and the National Cerebral and Cardiovascular Center who supported this study. We would like to thank Editage (www.editage.com) for English language editing.

### Author Contributions

Conceptualization: Hiroshi Kusunoki, Shotaro Tsuji, and Kazumi Ekawa; methodology: Hiroshi Kusunoki; software: Hiroshi Kusunoki, and Fumiki Yoshihara; validation: all authors; formal analysis: Hiroshi Kusunoki, Shotaro Tsuji, and Kazumi Ekawa; investigation: all authors; resources: Hiroshi Kusunoki, and Fumiki Yoshihara; data curation: Hiroshi Kusunoki, Nozomi Kato, and Keita Yamasaki; writing―original draft preparation: Hiroshi Kusunoki; writing―review and editing: Hiroshi Kusunoki, and Hideo Shimizu; visualization: Hiroshi Kusunoki; supervision: Hideo Shimizu; project administration: Hiroshi Kusunoki, and Hideo Shimizu; funding acquisition: none.

All authors have read and agreed to the published version of the manuscript.

### Approval by Institutional Review Board (IRB)

IRB approval number 111351 at Osaka Dental University.
